# Pharmacological evaluation of “Glyoherb”: A polyherbal formulation on streptozotocin-induced diabetic rats

**DOI:** 10.4103/0973-3930.60001

**Published:** 2010

**Authors:** Nima V. Thakkar, Jagruti A. Patel

**Affiliations:** Department of Pharmacology, Institute of Pharmacy, Nirma University of Science and Technology, Sarkhej-Gandhinagar Highway, Ahmedabad - 382 481, India

**Keywords:** Diabetes mellitus, glyoherb, hyperlipidemia, streptozotocin

## Abstract

In the present study, powdered suspension of ‘Glyoherb’- sugar control granules, a polyherbal formulation (manufactured by Dhanvantri Guj. herb., Valasan, Anand, Gujarat, India) was evaluated for its antihyperglycemic, antihyperlipidemic and antioxidant effects against normal and streptozotocin-induced diabetic rats. Type I diabetes was induced when streptozotocin 70 mg/kg was administered as a single i.p. injection. After five days of streptozotocin injection, animals showing glycosuria (fasting blood sugar level >200 mg/dl) were considered as diabetic. Daily oral administration of ‘Glyoherb’ suspension in 200, 400 and 600 mg/kg doses for 28 days produced a dose-dependant decrease in blood glucose levels. It also produced a significant decrease in elevated serum triglyceride, cholesterol, VLDL, LDL, atherogenic index, serum urea, and creatinine and in antioxidant parameters in a dose dependant manner. Results were analyzed using one way ANOVA followed by Tukey's test. No significant changes were noticed in blood glucose, serum lipid levels and kidney parameters in normal rats treated with ‘Glyoherb’ suspension alone. The efficacy of ‘Glyoherb’ suspension as an antihyperglycemic, antihyperlipidemic and antioxidant agent in streptozotocin-induced diabetes was comparable to that of the standard drug Glibenclamide (5 mg/kg).

## Introduction

Diabetes mellitus (DM), a leading metabolic disorder worldwide, is characterized by hyperglycemia associated with impairment in insulin secretion and/or insulin action as well as alteration in intermediary metabolism of carbohydrates, proteins and lipids. Several reports indicate that annual incidence rate of DM will increase in future worldwide, especially in India. It has been proposed that approximately 57 million Indians will be affected by DM by the year 2025.[1,2]

In patients with diabetes, coronary artery disease is the most common cause of death. Lipid abnormalities are commonly associated with diabetes. The most common lipid abnormalities in these patients include hypertriglyceridemia and reduced high-density lipoprotein (HDL) cholesterol levels. Although lipid abnormalities typically improve with better glycemic control, normalization does not usually occur. As a strong relationship exists between all forms of vascular disease in patients with diabetes and hyperlipidemia, it is important to screen for and treat these lipid abnormalities. Further, hyperlipidemia is partly responsible for the increased vascular disease as observed in patients with diabetes.

Diabetic nephropathy is the leading cause of DM- related morbidity and mortality. The pathogenesis of diabetic nephropathy is related to chronic hyperglycemia and hemodynamic alterations in renal microcirculation and structural changes in glomerulus.[3]

Synthetic hypoglycemic agents can produce serious side effects including hematological effects, hypoglycemic coma and disturbances of liver and kidney. In addition, they are not suitable for use during pregnancy.[4] Compared to synthetic drugs, herbal preparations are frequently considered to be less toxic with fewer side effects. Glyoherb is claimed to be a single unique formulation that provides a holistic management of blood glucose- and diabetes-related complications. Each 100 g of “Glyoherb”- sugar control granules (GH) contains: Gudmar Ext.: 15 gm; Mahamejva Ext.: 6 gm; Katuki Ext.: 6 gm; Chirata Ext.: 6 gm; Karela Ext.: 6 gm; Indrajav Ext.: 3 gm; Amla Ext.: 6 gm; Gokshur Ext.: 3 gm; Harde Ext.: 6 gm; Jambubij Ext.: 6 gm; Methi Ext.: 3 gm; Neem patti Ext.: 6 gm; Chanraprabha: 3 gm; Arogyavardhini: 3 gm; Harida Ext.: 3 gm; Bang bhasma: 3 gm; Devdar Ext.: 3 gm; as the major active constituents. In reducing the symptom of glycosuria, the dried leaves of Gudmar are being used in daily doses of 3-4 g for a period of three months or more. Bitter substances picrosides, bitter glycoside chiratin and bitter steroidal saponin, charantin are responsible for antidiabetic activity of Katuki, Chirata and Karela respectively. Amla contains the highest amount of vitamin C and other tannins responsible for antidiabetic action.

In the present study, attempts have been made to establish the scientific validity for the antidiabetic property of “Glyoherb”- sugar control granules, a polyherbal formulation using streptozotocin (STZ)-induced diabetic model in rats. The results of the study can serve as a step toward the development of an antihyperlipidemic herbal therapy for diabetes.

## Materials and Methods

### Drugs and chemicals

The polyherbal formulation ‘Glyoherb’- sugar contol granules was obtained from Dhanvantari Guj. Herb, Anand, Gujarat. These granules were suspended in 1% CMC solution for the preparation of an oral dosage formulation and administered by per oral (PO) route, in different doses, once daily for 28 days.

Streptozotocin was obtained from Sisco Pharmaceuticals Limited, Mumbai. Glibenclamide was obtained as a gratis sample from Zydus Cadila, Ahmedabad. All other chemicals used for the present study were of analytical grade. All diagnostic kits were procured from Lab-care diagnostics Ltd., India.

### Experimental animals

Wistar rats of either sex, 7-8 weeks old and weighing 150-250 g were used for the present study. The rats were randomly distributed to different groups consisting of six animals each. Experimental animals were maintained on standard pelleted laboratory animal feed and water *ad libitum*. Animals were maintained at 22 ± 2 C and 55 ± 5% relative humidity in a light controlled (12 h light/12 h dark) room. Animals that are described as fasting were deprived of food for at least 16 h but were allowed free access to drinking water. The protocol of the experiment (IPS/PCOL/MPH06/002) was approved by Institutional Animal Ethical Committee as per the guidance of the Committee for the Purpose of Control and Supervision of Experimentation on Animals (CPCSEA), Ministry of Social Justice and Empowerment, Government of India.

### Induction of diabetes

Rats were fasted overnight before being injected with STZ at a dose of 70 mg/kg of body weight (BW) (in cold 0.9% NaCl) via intraperitoneal (IP) route. The induction of diabetes was confirmed by determination of high fasting blood glucose level with polydipsia and polyuria on the fifth day of STZ administration. Rats with fasting blood glucose level >200 mg/dl were selected for experimentation.

### Experimental design

Rats were divided into seven groups, with six animals in each group. Group I: (NC), normal Control rats; fed with vehicle (1% CMC solution 2 ml, PO, 28 days); Group II: (only Glyoherb) normal rats treated with only Glyoherb suspension (600 mg/kg, PO, 28 days); Group III: (DC) diabetic control rats treated with vehicle (1% CMC Solution 2ml, PO, 28 days); Group IV (GC), diabetic rats treated with Glibenclamide (5 mg/kg, PO, 28 days); Group V, VI and VII (Glyoherb −200, Glyoherb −400, Glyoherb −600) diabetic rats treated with Glyoherb suspension (200, 400 and 600 mg/kg, PO, 28 days).

Fasting blood glucose was measured at different time intervals to check the hyperglycemic state. At the end of 28-days study period, blood samples were collected under fasting conditions; the serum was separated and was subjected to various biochemical estimations such as oral glucose tolerance test (OGTT), serum triglycerides (TG), high density lipoproteins (HDL) and cholesterol (CH). Very low density lipoprotein (VLDL) and atherogenic index were calculated using Friedewald's formula.[5]

### Biochemical estimations

Blood glucose was estimated by glucose oxidase-peroxidase (GOD/POD) method using commercially available diagnostic kits.[6] Serum cholesterol and triglycerides were measured by enzymatic colorimetric method[7,8] using commercially available diagnostic kits. Serum HDL was measured by precipitating reagent method.[9] Serum urea and creatinine were estimated by the modified Berthelot reaction method and without deproteinisation method[10,11] respectively using commercially available diagnostic kits. For OGTT glucose (1.5 g/kg/PO) was administered 90 min after pretreatment with respective drug solutions. Blood samples were withdrawn from retro-orbital plexus under light ether anesthesia at 0, 30, 60, 90 and 120 min of Glyoherb suspension or Glibenclamide administration. The serum obtained after centrifugation was used for the determination of glucose levels.

### Antioxidant activity in liver tissue homogenate

Liver was rinsed with ice cold distilled water followed by chilled sucrose solution (20%). About 1 g of liver tissue was homogenized in 10 ml of ice cold Tris hydrochloride buffer. The prepared liver tissue homogenates were centrifuged at 3500 rpm for 15 min. and supernatant was used for the determination of various antioxidant parameters like tissue proteins, malondialdehyde (MDA) levels, reduced glutathione (GSH) levels, superoxide dismutase (SOD) and catalase.[12–15]

### Statistical analysis

Results are expressed as mean ± SEM and statistical difference was evaluated using one-way analysis of variance (ANOVA) followed by Tukey's test. Data were considered statistically significant at *P* value ≤0.01 and highly significant at *P* < 0.001. Statistical analysis was performed using Graph Pad statistical software.

## Results

### Effect of Glyoherb on serum glucose levels

Streptozotocin treatment produced significant increase in serum glucose levels (319.98 ± 34.14 mg/dl) with respect to the control group (87 ± 3.14 mg/dl). The hyperglycemia was pronounced after five days. As shown in Table 1, the administration of Glyoherb suspension (600 mg/ kg) or Glibenclamide (5 mg/kg) significantly reversed (80.24 ± 7.37 mg/dl and 99.40 ± 4.98 mg/dl respectively) the increase in serum glucose concentration induced by STZ. Such effect was more obvious with the high dose of Glyoherb 600 mg/kg body weight.

**Table 1:** Effect of Glyoherb on serum glucose levels and oral glucose tolerance test in control and STZ-induced diabetic rats (*n* = 6)

Groups	Parameters		
	
	Serum glucose levels (mg/dl)	Oral glucose tolerance test	
		
		0 time glucose levels (mg/dl) 120 min glucose levels (mg/dl)	
Normal control rats	87 ± 3.14	70.46 ± 14.32	90.15 ± 11.46
Normal rats treated with only Glyoherb suspension	57.38 ± 1.47	47.38 ± 5.38	70.91 ± 8.47
Diabetic control rats	319.98 ± 34.14*	292.66 ± 18.37*	506.58 ± 38.89*
Glibenclamide (5 mg/kg, PO, 28 days)	99.4 ± 4.98#	87.09 ± 15.58#	160.55 ± 45.16#
Glibenclamide (5 mg/kg, PO, 28 days)	170.82 ± 20.02#	193.32 ± 4.91#	258.05 ± 16.56#
Glyoherb suspension (400 mg/kg, PO, 28 days)	102.91 ± 7.12#	72.34 ± 8.54#	157.47 ± 33.63#
Glyoherb suspension (600 mg/kg, PO, 28 days)	80.24 ± 7.37#	96.15 ± 4.92#	222.31 ± 23.67#

Values are expressed as mean ± S.E.M;

* -Significantly different from normal control (*P* < 0.05);

# -Significantly different from diabetic control (*P* < 0.05)

### Effect of Glyoherb on oral glucose tolerance test

Administration of glucose (1.5 g/kg, PO) did not produce any significant change in the serum glucose levels of either control (90.15 ± 11.46 mg/dl) or only Glyoherb-treated group animals (70.91 ± 8.47 mg/ dl) even at the end of 2 h [Table 1]. However, oral glucose administration, to diabetic control group animals markedly increased their glucose levels (506.58 ± 38.89 mg/dl). Serum glucose levels of diabetic rats treated with Glyoherb suspension in the dose of 200, 400 and 600 mg/ kg or Glibenclamide were significantly decreased (258.05 ± 16.56, 157.47 ± 33.63, 222.31 ± 23.67 and 160.55 ± 45.16 mg/dl respectively) at the end of 120 min when compared with the diabetic control.

### Effect of Glycoherb on lipid profile

STZ produced significant increase in serum triglycerides (127.84 ± 10.31 Vs 64.65 ± 5.76 mg/dl in normal control rats), serum cholesterol (226.65 ± 11.57 Vs 93.53 ± 2.7 mg/dl in normal control rats), LDL cholesterol (159.67 ± 23.28 Vs 100.1 ± 3.71 mg/dl in normal control rats), VLDL cholesterol (33.89 ± 3.56 Vs 13.18 ± 1.76 mg/dl in normal control rats) and atherogenic index (5.26 ± 1.33 Vs 0.86 ± 0.24 in normal control rats), as well as marked reduction in serum HDL levels (24.75 ± 1.24 Vs 49.19 ± 2.68 mg/dl in normal control rats). As shown in Table 2 treatment with Glyoherb 600 mg/kg reduced serum cholesterol, triglycerides, VLDL, LDL levels and atherogenic index (146.63 ± 8.24, 77.29 ± 15.58, 20.52 ± 3.39, 98.83 ± 7.93 mg/dl and 1.91±1.5 respectively) which was comparable to control group (93.53 ± 2.7, 64.65 ± 5.76, 13.18 ± 1.76, 100.1 ± 3.71 mg/dl and 0.86 respectively). The standard drug Glibenclamide also produced the same effect (167.48 ± 8.03, 95.51 ± 9.84, 19.23 ± 1.98, 109.52 ± 3.67 mg/dl and 1.74 ± 0.8 respectively). Increase in HDL levels were also much pronounced in animals treated with Glyoherb 400 mg/kg or Glibenclamide 5 mg/kg (59.27 ± 17.23 and 54.21 ± 7.14 mg/dl) which was comparable to normal control animals (49.19 ± 2.68 mg/dl).

**Table 2:** Effect of Glyoherb on lipid profile in control and STZ-induced diabetic rats (*n* = 6)

Groups	Parameters					
	
	Serum cholesterol (mg/dl)	HDL cholesterol (mg/dl)	Serum triglycerides (mg/dl)	LDL cholesterol (mg/dl)	VLDL cholesterol (mg/dl)	Atherogenic index
Normal control rats	93.53 ± 2.7	49.19 ± 2.68	64.65 ± 5.76	100.1 ± 3.71	13.18 ± 1.76	0.86 ± 0.24
Normal rats treated with only Glyoherb suspension	101.7 ± 8.49	45.82 ± 2.77	52.98 ± 9.63	106.86 ± 6.79	12.39 ± 1.89	0.74 ± 0.27
Diabetic control rats	226.65 ± 11.57*	24.75 ± 1.24*	127.84 ± 10.31*	159.67 ± 23.28*	33.89 ± 3.56*	5.26 ± 1.33*
Glibenclamide (5 mg/kg, PO, 28 days)	167.48 ± 8.03#	54.21 ± 7.14#	95.51 ± 9.84	109.52 ± 3.67#	19.23 ± 1.98#	1.74 ± 0.8#
Glibenclamide (5 mg/kg, PO, 28 days)	180.41 ± 6.04#	28.35 ± 10.37	120.13 ± 12.25	118.31 ± 6.27	20.79 ± 3.41#	4.14 ± 2.17
Glyoherb suspension (400 mg/kg, PO, 28 days)	147.3 ± 8.25#	59.27 ± 17.23#	103.96 ± 17.06	110.01 ± 10.23#	18.73 ± 1.6#	2.06 ± 0.61#
Glyoherb suspension (600 mg/kg, PO, 28 days)	146.63 ± 8.24#	40.82 ± 11.98	77.29 ± 15.58#	98.83 ± 7.93#	20.52 ± 3.39#	1.91 ± 1.5#

Values are expressed as mean ± S.E.M;

* -Significantly different from normal control (*P* < 0.05);

# -Significantly different from diabetic control (*P* < 0.05)

### Effect of Glyoherb on kidney parameters

**Figure 1:**
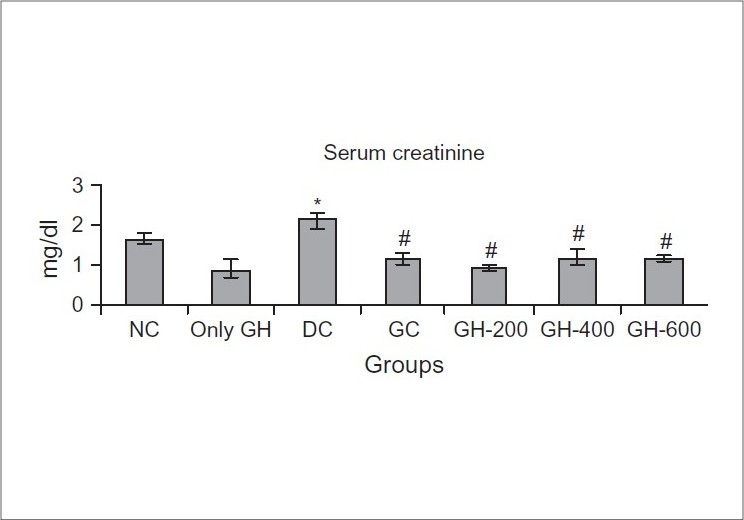
Effect of Glyoherb on serum creatinine levels in control and STZ induced diabetic rats (*n* = 6); Values are expressed as Mean ± S.E.M.; Statistical evaluation was done using ANOVA followed by Tukey's test.; *-Significantly different from normal control (*P* < 0.05); #-Significantly different from diabetic control (*P* < 0.05)

**Figure 2:**
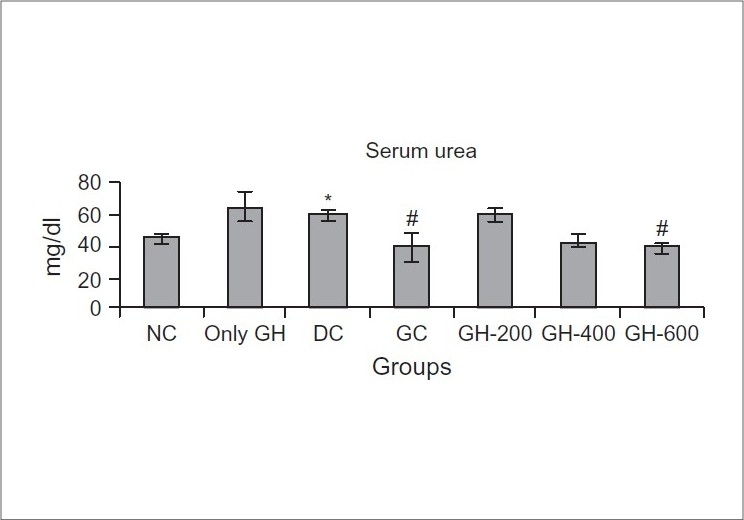
Effect of Glyoherb on serum urea levels in control and STZ induced diabetic rats (*n* = 6); Values are expressed as Mean ± S.E.M; Statistical evaluation was done using ANOVA followed by Tukey's test; *-Significantly different from normal control (*P* < 0.05); #-Significantly different from diabetic control (*P* < 0.05)

STZ-diabetic rats exhibited higher serum creatinine (2.15 ± 0.18 mg/dl) and urea (58.88 ± 3.37 mg/dl) levels as compared to those of normal control rats (1.67 ± 0.097 and 44.73±2.22 mg/dl) [Figures 1 and 2]. Chronic treatment with ‘Glycoherb’ sugar control granules (600 mg/kg) significantly reduced the elevated creatinine as well as urea levels in diabetic rats (1.19 ± 0.11 and 38.49 ± 4.29 mg/dl), which was comparable to that of Glibenclamide treated animals (1.15 ± 0.17 and 38.81 ± 8.67 mg/dl) [Figures 1 and 2].

### Effect on antioxidant parameters in liver

The STZ-induced diabetic rats (DC) showed a significant decrease in SOD, catalase (3.66 ± 0.39 and 4.24 ± 0.81 units/min/mg protein) and GSH levels (40.35 ± 11.59 mg/gm protein) when compared with the normal control animals (7.61 ± 0.49; 11.88 ± 2.65 units/min/mg protein and 107.31 ± 8.77 mg/ gm protein respectively). The TBARS significantly increased following STZ administration (21.77 ± 2.05 nmoles/mg protein) in DC rats when compared to normal control rats (10.69 ± 0.17 nmoles/mg protein). Administration of Glycoherb suspension in various doses for 28 days produced a marked increase in antioxidant (SOD and Catalase levels) whereas it produced a significant decrease in prooxidant parameter (TBARS) [Table 3].

**Table 3:** Effect of Glyoherb on antioxidant parameters in control and STZ induced diabetic rats (*n* = 6)

Group	Parameters			
	
	SOD (units/min/mg protein)	Catalase (units/min/mg protein)	TBARS (nmoles/mg protein)	GSH (mg/gm protein)
Normal control rats	7.61 ± 0.49	11.88 ± 2.65	10.69 ± 0.17	107.31 ± 8.77
Normal rats treated with only Glyoherb suspension	6.29 ± 0.47	10.98 ± 2.02	9.88 ± 0.19	128.68 ± 10.1
Diabetic control rats	3.66 ± 0.39*	4.24 ± 0.81*	21.77 ± 2.05*	40.35 ± 11.59*
Glibenclamide (5 mg/kg, PO, 28 days)	4.91 ± 0.18#	8.76 ± 1.83	10.19 ± 0.64#	69.01 ± 8.13
Glibenclamide (5 mg/kg, PO, 28 days)	4.25 ± 0.07	1.51 ± 0.51	8.49 ± 0.76#	61.52 ± 11.74
Glyoherb suspension (400 mg/kg, PO, 28 days)	5.92 ± 0.21#	13.39 ± 3.36#	8.7 ± 1.74#	59.86 ± 5.64
Glyoherb suspension (600 mg/kg, PO, 28 days)	4.85 ± 0.25#	8.62 ± 1.91	8.45 ± 1.55#	56.67 ± 5.29

Values are expressed as mean ± S.E.M;

* -Significantly different from normal control (*P* < 0.05);

# -Significantly different from diabetic control (*P* < 0.05)

## Discussion

Streptozotocin (STZ), a β-cytotoxin, induces ‘chemical diabetes’ in a wide variety of animal species including rat by selectively damaging the insulin-secreting β-cells of the pancreas. Intraperitoneal injection of STZ produces fragmentation of DNA of β-cells of pancreas which stimulates poly (ADP-ribose) and depletes NAD ultimately leading to destruction of β-cells and it is evidenced by clinical symptoms of hyperglycemia and hypoinsulinaemia.[16,17] The serum glucose, lipid and cholesterol values for the rats are in agreement with those expected for streptozotocin diabetic rats.[18,19]

STZ-diabetes in type 1 diabetics produce a significant increase in glucose levels associated with decrease in insulin levels.[20] Treatment with polyherbal formulation ‘Glyoherb’ sugar control granules showed significant decrease in fasting serum glucose levels which was near to healthy control. The antidiabetic plant extracts may involve one or more compounds which decreases blood glucose levels suggesting that the natural constituents could act synergistically to induce a hypoglycemic effect as described by Marles and Fransworth.[21–23]

Decrease in blood sugar levels was found to be more effective with ‘Glyoherb’ in the dose of 400 and 600 mg/kg. The test formulation showed dose dependant effects. Glibenclamide showed rapid normalization of blood glucose due to its insulin releasing effects.

At the end of treatment schedule (i.e. after 28 days) animals were subjected to oral glucose tolerance test (OGTT) which directly measures the action of endogenous insulin in response to a glucose stimulus.[24] However, this method does not allow a separate evaluation of β islet cells and peripheral insulin sensitivity of the tissues. Our data suggest that diabetic animals have higher levels of glucose after 120 min of glucose load. Lack of or reduced levels of insulin may be responsible for this observation. However, diabetic animals treated with ‘Glyoherb’ or ‘Glibenclamide’ show better tolerance to oral glucose load.

Earlier studies have shown that in STZ-induced diabetic rats, insulin deficiency is associated with hypercholesterolemia and hypertriglyceridemia. Insulin deficiency may be responsible for dyslipidemia, because insulin has an inhibitory action on HMG-CoA reductase, a key enzyme that is rate limiting in the metabolism of cholesterol rich LDL particles.[25] The mechanisms responsible for the development of hypertriglyceridemia in uncontrolled diabetes in humans (possibly in insulin deficient STZ-diabetic rats) may be due to a number of metabolic abnormalities that occur sequentially. Acute insulin deficiency initially causes an increase in free fatty acid mobilization from adipose tissue, resulting in increased secretion of VLDL-triglyceride from liver.[26] In diabetic rats, there is a decrease in lipoprotein lipase activity[27] resulting in impaired clearance of VLDL and chylomicrons from plasma.[28] In our study, treatment with ‘Glycoherb’ granules suspended in 1% CMC solution significantly decreased both serum cholesterol and triglyceride levels in diabetic rats. There were also significantly decreased serum LDL and VLDL levels by treatment with ‘Glyoherb’ compared to diabetic control animals. It also produced significant change in serum HDL level by treatment compared to diabetic control group. Glibenclamide-treated animals have also beneficial effects on lipid profile.

The morphological abnormalities in type 1 diabetic rats were associated with a significant elevation in serum creatinine and urea levels indicating impaired renal function of diabetic animals. Chronic treatment with ‘Glyoherb’ granules for 28 days significantly decreased elevated serum creatinine and urea level in diabetic rats, which indicated its beneficial effects on kidney.

Oxidative stress plays a major role in the pathogenesis of both types of diabetes mellitus. Free radical production caused by hyperglycemia may occur via at least three different routes: Nonenzymatic glycation,[29] auto-oxidation of glucose and intracellular activation of the polyol pathway.[30–32] High levels of free radicals and simultaneously declined antioxidant enzyme levels lead to cell damage, inactivation of enzymes and lipid peroxidation. In the present study, diabetic control rats showed significant increase in TBARS levels and decrease in SOD, catalase and glutathione levels in the rat liver homogenates compared to normal rats, indicating a dysfunction in antioxidant defensive system in diabetes mellitus. Treatment with ‘Glyoherb’ suspension in all the doses reduced double fold the TBARS levels compared to diabetic controls. On the other hand, it increased SOD and catalase levels in rat liver homogenates compared to diabetic rats, which were comparable to that of Glibenclamide-treated animals. This antioxidant activity of ‘Glyoherb’ sugar control granules in IDDM rats may be due to the presence of Gudmar, Karela, Katuki, Amla and Mahamejva. All these constituents have well reported antidiabetic action. The main constituents responsible for antidiabetic action may be gymnemic acid from gudmar, picrosides from Katuki, olephic acid, chirantin from Chirata and various steroidal saponins from karela.[33,34]

## Conclusion

Our data suggest that ‘Glyoherb’ sugar control granules possess potential antidiabetic activity as it lowers serum glucose levels and increases glucose tolerance in STZ-induced type 1 diabetic rats. This polyherbal formulation also possess significant antihyperlipidemic activity as it lowers serum cholesterol and triglyceride levels. ‘Glyoherb’ did not exert any toxic effects in STZ-induced impaired kidney and liver functions. It was rather found to be improving kidney and liver functions. In addition, ‘Glyoherb’ possesses potential antioxidant activity as it decreases lipid peroxidation and enhances antioxidant status in diabetic rats. The antidiabetic activity of ‘Glyoherb’ may be attributed to its antioxidant properties also. Thus, data from the present study indicate antidiabetic, antihyperlipidemic and antioxidant properties of ‘Glyoherb’- a poly herbal formulation against STZ-induced type 1 diabetes in wistar rats. Hence, ‘Glyoherb’ may be regarded as a promising natural and safe remedy for the prevention or delay of diabetic complications.
